# An approach to and the rationale for the pharmacological management of behavioral and psychological symptoms of dementia

**DOI:** 10.4103/0972-2327.74252

**Published:** 2010-12

**Authors:** Manjari Tripathi, Deepti Vibha

**Affiliations:** Department of Neurology, AIIMS, New Delhi, India; 1 Department of Neurology, Institute of Liver and Biliary Sciences, New Delhi, India

**Keywords:** Behavior, dementia, diagnosis, psychological, therapeutics

## Abstract

The behavioral and psychological symptoms of dementia (BPSD) have been a difficult management area for neurologists and psychiatrists alike. The correct identification of each symptom and the underlying precipitating cause is the key to proper management—nonpharmacological as well as pharmacological. BPSD has been well documented in all types of dementia in various stages of the disease and in all dementias at an advanced stage. The proper management is not only rewarding in terms of responsiveness in an otherwise “incurable” and progressive disease, but also improves the quality of life of the patients and the caregivers alike. The caregiver burden is greatly decreased by an efficient management of BPSD. This review discusses the implications and boundaries of the term BPSD and unravels each symptom and its identification. Manifestations of psychological symptoms such as delusion, hallucination, misidentification, psychosis, depression, apathy, and anxiety are briefly described. Correct identification of behavior symptoms such as wandering, agitation, catastrophic reaction, disinhibition, and delirium has been outlined. While the subtle differences in each entity make the precise identification difficult, the different therapeutics of each make the exercise necessary. Pharmacological recommendations and side effects of medications have been mentioned thereafter. The review will help in the identification and correct pharmacological management of BPSD.

## Introduction

With the aging of the world’s population, there is a projected increase in the absolute number of elderly with Alzheimer’s disease (AD) and other irreversible dementias. Dementia is associated with progressive cognitive disability, a high prevalence of behavioral and psychological symptoms of dementia (BPSD), such as agitation, depression and psychosis, stress in caregivers, and costly care. Projections for 2025 are that the number of people with dementia will reach 34 million worldwide and 25 million in developing countries. The term BPSD was proposed in 1994, which the International Psychogeriatric Association (IPA) elucidated and later improvised on this concept.[1] The good news is that mostly BPSD are treatable and generally respond better to therapy than any other symptoms or syndromes of dementia. Treatment of BPSD offers the best chance to alleviate suffering, reduce family burden, and lower societal costs in patients with dementia. BPSD have been well documented among patients with Alzheimer’s disease (AD), vascular dementia (VaD), progressive supranuclear palsy (PSP), Parkinson’s disease (PD), frontotemporal dementia (FTD), and more recently mild cognitive impairment (MCI).[2–6] The consensus group, consisting of some 60 experts in the field, from 16 countries, produced a statement on the definition of the BPSD: “The term behavioral disturbances should be replaced by the term behavioral and psychological symptoms of dementia (BPSD), defined as symptoms of disturbed perception, thought content, mood, or behavior that frequently occur in patients with dementia.”[7]

### What are the behavior and psychological symptoms found?

Dementia patients often present with a wide array of neuropsychiatric symptoms such as psychosis and mood disturbance (e.g., depression, apathy, hallucinations, and delusions), alterations in behavior (e.g., aggression, rummaging, and pacing), and alterations in biological functions (e.g. changes in eating and sleeping habits).

For the purpose of classification and simplification, the symptoms may be divided into behavioral and psychological symptoms [Table 1].

**Table 1:** Behavior and psychological symptoms of dementia

Behavioral symptoms	Psychological symptoms
Usually identified on the basis of observation of the patient, including physical aggression, screaming, restlessness, agitation, wandering, culturally inappropriate behavior, sexual disinhibition, hoarding, cursing, and shadowing.	Usually and mainly assessed on the basis of interviews with patients and relatives; these symptoms include anxiety, depressive mood, hallucinations, and delusions.

BPSD symptoms can occur in any stage of dementia, but do vary in frequency and timing of presentation according to the type of dementia. These symptoms not only impair functional performance, but also lead to increased caregiver burden, increased cost of treatment, and more frequent institutionalization.[8]

One study of BPSD found that 64% of patients with AD had one or more BPSD at initial evaluation.[9] In a community-based population survey using the neuropsychiatric inventory (NPI), Lyketsos *et al*.[10] reported that people with dementia had over 40 times the rate of behavioral disturbance than rest of the population. They also reported that delusions are more frequent in AD, and there is a higher rate of depression in VaD. In another study by Cohen *et al*.,[11] patients with mixed AD and VaD had highest levels of psychiatric disturbance. Visual hallucinations are more commonly found in people with dementia with Lewy bodies than in those with AD or PD.[12] Fronto-temporal dementia has been associated with higher incidences impulsivity, compulsive behaviors, hypersexuality, and verbal outbursts.[13,14]

## Specific Symptoms: Psychological

### Delusions

The frequency of delusion varies between 10% and 73%.[15] The most common delusions in demented people are persecutory or paranoid. Broadly, five types of delusions have been described [Table 2].[16]

**Table 2:** Delusion types seen in dementia

Delusion	Comments
People stealing things	If the delusion is severe the demented person will believe that others are coming into the home to hide or steal objects.
House is not one’s home (misidentification)	The patient no longer remembers or recognizes his/her home. This results in wandering.
Spouse (or other caregiver) is an impostor–Capgras phenomenon	Can provoke anger or violence toward the perceived impostor. Contributes to increased caregiver burden.
Abandonment	The individual’s awareness of having become a burden may be related to this delusion of abandonment
Infidelity	Persons with dementia will become convinced that their spouse is unfaithful–sexually or otherwise.

### Hallucinations

Hallucinations should be differentiated from visual agnosia and misidentification due to poor vision. The frequency of hallucinations in dementia ranges from 12% to 49%.[17] Visual hallucinations are the most common occurring in up to 30% of patients, and these are more common in moderate dementia. In people with Lewy bodies, the frequency can be as high as 80%.[18]

### Misidentifications

Misidentifications in dementia are examples of disorders of perception.[19]

They can be of four types:

- Presence of persons in the patient’s own house (the “phantom boarder” syndrome)
- Misidentification of the patient’s own self (often seen as not recognizing their own mirror reflection)
- Misidentification of other persons
- Misidentification of events on television (the patient imagines these events are occurring in a real three-dimensional space)

Delusional misidentification syndromes described earlier, e.g. Capgras syndrome can be very distressing and difficult to manage.

### Psychosis

The occurrence of psychosis in dementia calls for early institutionalization. However associated mood disorder, occurrence with concomitant delirium, or precipitation with drugs or medication might complicate the picture. With dementia, however, eventual remission occurs and antipsychotics can be tapered off.

### Depression

Depressed mood occurs in 40–50% of patients of dementia.[15] Diagnosing depression can be difficult, particularly in patients with moderate and severe dementia. Depressive disorder should, therefore, be considered when one or more of the following conditions are noted[20]:

- A pervasive depressed mood and loss of pleasure
- Self-deprecatory statements and expressed wishes to die
- A family or personal history of depression prior to the onset of dementia.

### Apathy

Apathy is present in up to 50% of patients in the early and intermediate stages of AD and other dementias. Patients who are apathetic show a lack of interest in daily activities and personal care with decreased social interaction, poor facial expression, decreased emotional responsiveness, and lack of initiative. These symptoms may be easily mistaken for those of major depression. Although lack of motivation occurs in apathy and depression, the syndrome of apathy denotes lack of motivation without the dysphoria or vegetative symptoms of depression. The clinician must distinguish a patient who is apathetic from one who is depressed, since the management of each disorder differs. For example, on a pharmacological basis, a patient with depression may require antidepressant medication, while another with apathy may benefit from a cholinesterase inhibitor.

### Anxiety

Patients with anxiety express worries about previously nonstressful events and activities like being away from home. A person with Godot syndrome repeatedly asks questions about an upcoming event—a behavior which appears to result from decreased cognitive (specifically memory) abilities and from the inability to channel remaining thinking capacities productively. This can become as incessant and persistent as to create a major burden for the patient’s family and caregivers.[21]

## Specific Symptoms: Behavioral

### Wandering

There are several different types of behavior covered by the term “wandering”[22]:

- Checking (repeatedly seeking the whereabouts of the carer or occasionally another person)
- Trailing or stalking (an extreme form of checking—following the caregiver or another person around excessively)
- Pottering or rooting (walking around the house or gardening trying ineffectively to carry out tasks (e.g., washing/drying up, cleaning, and weeding)
- Aimless walking
- Night-time walking
- Walking directed toward an inappropriate purpose
- Excessive activity
- Wandering off, needed to be brought back to the house
- Repeatedly attempting to leave the house

This may be due to hyperactivity or a faulty navigational ability.

### Agitation

Agitation is defined as inappropriate verbal, vocal, or motor activity that is not judged by an outside observer to result directly from the needs or confusion of the person.[23] Medical, psychological, and environmental factors and premorbid personality have consistently been shown to affect agitation.

### Catastrophic reactions

Catastrophic reactions, sometimes referred to as rage reactions, are characterized by an excessive and sudden emotional response or physical behavior. Catastrophic reactions present as sudden angry outbursts, verbal aggression (e.g., shouting and cursing), threats of physical aggression, and physical aggression (e.g., hitting, kicking, and biting). They can be considered as an outcome of psychological disturbances.

### Disinhibition

Patients with disinhibition syndrome behave impulsively and inappropriately. Other symptoms associated with disinhibition include crying, euphoria, and verbal aggression, physical aggression toward other persons and objects, self - destructive behavior, sexual disinhibition, motor agitation, intrusiveness, impulsiveness, and wandering.

### Delirium

Despite their similarities, it is usually possible to differentiate between delirium and dementia because delirium usually presents with acute or subacute onset of symptoms, heightened or reduced attention in a patient with pre - existing dementia, prominent fluctuations in symptoms, and visual hallucinations accompanied by agitation. Other signs of delirium include altered psychomotor activity or asterixis. Delirium is usually precipitated by infection, malnutrition, dehydration, metabolic disturbances, drugs, or surgery.

### Pharmacological management of behavioral and psychological symptoms of dementia

It should be emphasized at the beginning that nonpharmacological management plays a crucial role in BPSD and medications have to be judiciously used in elderly and demented patients with comorbidities. The underlying precipitating cause should not be missed. Therefore, the first step in the management involves the careful assessment and correction of any physical, psychosocial or environmental triggers, or perpetuating factors in the genesis of BPSD. Drug treatment for BPSD should be time limited and, with the exception of antidepressant treatment for depression, should not exceed 12 weeks without a review of the treatment regimen. When medication is discontinued, however, it is possible that some patients will experience a recurrence of symptoms, in which case medication should be reinstated. Recommendations for treatment of various symptoms have recently been published and are summarized in Table 3.[24]

It goes beyond saying to state that the side effects of each pharmacological agent should be known lest these are confused with worsening symptoms or nonresponsiveness.

Table 4 lists the common side effects of above-mentioned pharmacological agents.[26]

**Table 3:** Treatment recommendations and doses

Treatments for agitation and hallucinations/delusions associated with AD			
Recommendation Grade A Risperidone started at 0.5 mg/day (may be increased to 2 mg/day) Olanzapine started at 2.5 mg/day (may be increased to 10 mg/day) Quetiapine started at 25 mg/day (may be increased to 100 mg/day) Aripiprazole started at 2 mg/day (may be increased to 15 mg/day)			
Recommendation Grade B Tiapride started at 50–100 mg/day (may be increased to 300 mg/day) Haloperidol started at 0.75 mg/day (may be increased to 2–3 mg/day) If the above medications are ineffective: Carbamazepine started at 100–300 mg/day (may be increased to 600 mg/day)			
Recommendation Grade C Sodium valproate, trazodone, SSRIs, etc.			

**Drug**	**Start range (mg)**	**Dose (mg)**	**Schedule**

Haloperidol	0.5	0.5–2	Once daily
Thiothixene	1	1–10	Once daily
Risperidone	0.5	0.5–2	Once daily
Clozapine	6.25	10–100	Twice or once daily
Olanzapine	2.5	5–10	Once daily
Quetiapine	25	25–150	Divided doses

**Drug**		**Initial dose (mg)**	**Target dose (mg)**

Paroxetine		10	20–30
Fluoxetine		10	20–30
Sertraline		25	50–100
Nortriptyline		10	20–60
Moclobemide		150	150–600
Mirtazepine		15	15–45

**Treatments for depression**
Recommendation Grade B
SSRIs
**Treatments for delirium**
Recommendation Grade C (but close to Grade B)
Risperidone started at 0.5–1.0 mg/day Risperidone started at 0.5–1.0 mg/day Olanzapine started at 2.5–5 mg/day Tiapride started at 25–50 mg/day
**Treatments for sleep disorders**
Recommendation Grade B
Zolpidem 5–10 mg/day
Recommendation Grade C (but close to Grade B)
Benzodiazepine sleep inducers (short-acting, intermediate-acting) Trazodone and antipsychotics

**Table 4:** Side effects of various therapeutic agents

Drug	Side effects
Typical neuroleptics	Extrapyramidal side effects (EPS) (e.g., drooling, rigidity, and akinesia) with high-potency conventional agents such as haloperidol and thiothixene Postural hypotension and anticholinergic side effects (e.g., dry mouth, constipation, blurred vision, urinary hesitancy and retention, increased confusion) with low-potency conventional agents such as thioridazine and chlorpromazine.
Newer antipsychotics	Have lower propensity to cause EPS, may be less likely to cause tardive dyskinesia. Clozapine has significant anticholinergic and postural hypotensive effect. It is association with a risk of agranulocytosis and requires weekly white cell count monitoring.
Benzodiazepines	Excessive sedation (drowsiness), ataxia, amnesia, and confusion. Increased risk of falls in dementia.[25]
Carbamazepine	Sedation, skin rash, headache, and mild elevation of liver function tests. Overdosing causes ataxia
Valproic acid	Sedation, diarrhea, tremor, nausea, weight gain, hair loss, and abnormal liver function
Tricyclic antidepressants	Postural hypotension, blurred vision, urinary hesitancy, and intracardiac conduction defects.
Selective serotonin reuptake inhibitors	Less common and severe side effects than TCAs Gastrointestinal symptoms (e.g., nausea, vomiting), akathisia, restlessness, insomnia, weight loss, hyponatremia

## Conclusion

The cardinal symptoms of behavior and psychological problems in dementia need to be identified for proper management. While nonpharmacological therapy also plays a cardinal role, pharmacological management is rewarding. While dementia is relentlessly progressive despite the recent armamentarium of disease modifying drugs, what distress most to the patient and caregiver are the behavioral problems. It is important to have a sound knowledge of the medicines used, their doses, duration, and side effects to be known for improving the quality of life.
